# An Unexpected Property of Adhesive Gold

**Published:** 1874-05

**Authors:** Thomas Fletcher


### ARTICLE IV.

### *An Unexpected Property of Adhesive Gold.*

BY THOMAS FLETCHER, ESQ., F. C. S.

Having slowly come to the conclusion that adhesive foil, sponge gold, &c., although easy to work, do not give in all cases thoroughly permanent results, I endeavoured to find a satisfactory reason why one peculiar character of gold, apparently so good, should with me prove in practice distinctly worse than the old-fashioned foil with little or no adhesiveness.

Amongst other experiments I applied my own tube test to different samples of gold, and the results were so strange and unexpected that I repeated them time after time with the greatest care before I could convince myself that the results were always the same and were not caused by carelessness or oversight in any way.

Taking a strong glass tube, quarter inch bore, three quarters inch long, I partially closed one end with the blow-pipe, and in the small hole left, carefully anchored an adhesive gold plug, building it up with the greatest possible care, using in some cases a Snow and Lewis mallet, and in others hand pressure only. After making repeated trials with adhesive foil, sponge, plastic gold, annealed cylinders, and blocks, using every care, I failed totally in making one single plug tight against moisture. Pack as I would, the filling up of the vacant part of the tube with a coloured solution was speedily followed by the penetration of the fluid by capillary action between the glass and the plug.

When I used soft, non-adhesive gold or tin foil there was no difficulty, and every plug was absolutely tight. The plugs of adhesive gold were apparently a perfect fit, showing no inperfections under a powerful magnifying glass, but the unpleasant fact still remained, that not one was tight enough to resist the passage of moisture down its sides, spite of attempts to wedge the plugs by the use of conical points. Whilst testing some samples of amalgam recently, I was surprised to see some plugs which were very perfect in my hands a few minutes before had become suddenly very faulty. After some experiments I found that the heat of my hands had in a few minutes caused sufficient expansion of the glass to allow the coloured solution to pass between the glass and the plug, and that a large plug in a very smooth glass tube could be made quite loose by the heat of the hand alone.

It is possible that the alternate expansion and contraction of a rigid mass of adhesive gold or amalgam may have something considerable to do with the ultimate failure where the plug is large and much exposed to contact with food at different temperatures. This cause of failure would not be likely to exist with non-adhesive foil, wedged in position, as it always will retain a certain amount of elasticity.

The question raised as to adhesive foil and sponge gold by these experiments is one of such importance that it should not be allowed to rest on the conclusions of any one operator, as the manner of working varies so greatly, and it also remains to be seen whether the process of testing in glass tubes is one which will in all cases bear out the practical results in the mouth. The reason glass was chosen is that faults may so readily be seen and the packing can be carried on in a much more perfect manner, whilst the plug and the point of the instrument can be watched closely.

Since writing the above I have attempted to make watertight plugs of adhesive foil and sponge gold in cavities in ivory with exactly the same results, and still not being satisfied I gave the result of my experience to two of the best operators I know. The reply in each case was, "Oh, I will make a tight plug for you," the remark being accompanied with a "superior" smile which I fully appreciated. Neither of these tight plugs have come to hand as yet, and it is pretty evident that the failure of adhesive gold in my hands is not an exceptional case. I have now tried some seven or eight different forms of adhesive gold with the same results invariably, and have failed with a foil after annealing which made a sound and tight plug before. When soft and adhesive gold are used alternately, the fluid penetrates to the adhesive part only, being unable to penetrate past a layer of soft foil unless carelessly inserted.

I forgot to mention that the plugs in ivory were examined by being sawn through, after being covered with solution for a time and afterwards dried.—*British Journal of Dental Science.*

